# Bacteriophage: A new therapeutic player to combat neutrophilic inflammation in chronic airway diseases

**DOI:** 10.3389/fmed.2022.1069929

**Published:** 2022-12-14

**Authors:** Daniel R. Laucirica, Stephen M. Stick, Luke W. Garratt, Anthony Kicic

**Affiliations:** ^1^Wal-yan Respiratory Research Centre, Telethon Kids Institute, The University of Western Australia, Nedlands, WA, Australia; ^2^Department of Respiratory and Sleep Medicine, Perth Children’s Hospital, Nedlands, WA, Australia; ^3^Centre for Cell Therapy and Regenerative Medicine, School of Medicine and Pharmacology, The University of Western Australia and Harry Perkins Institute of Medical Research, Nedlands, WA, Australia; ^4^School of Population Health, Curtin University, Bentley, WA, Australia

**Keywords:** bacteriophage, phage therapy, chronic airway disease, bacterial infection, neutrophilic inflammation

## Abstract

Persistent respiratory bacterial infections are a clinical burden in several chronic inflammatory airway diseases and are often associated with neutrophil infiltration into the lungs. Following recruitment, dysregulated neutrophil effector functions such as increased granule release and formation of neutrophil extracellular traps (NETs) result in damage to airway tissue, contributing to the progression of lung disease. Bacterial pathogens are a major driver of airway neutrophilic inflammation, but traditional management of infections with antibiotic therapy is becoming less effective as rates of antimicrobial resistance rise. Bacteriophages (phages) are now frequently identified as antimicrobial alternatives for antimicrobial resistant (AMR) airway infections. Despite growing recognition of their bactericidal function, less is known about how phages influence activity of neutrophils recruited to sites of bacterial infection in the lungs. In this review, we summarize current *in vitro* and *in vivo* findings on the effects of phage therapy on neutrophils and their inflammatory mediators, as well as mechanisms of phage-neutrophil interactions. Understanding these effects provides further validation of their safe use in humans, but also identifies phages as a targeted neutrophil-modulating therapeutic for inflammatory airway conditions.

## Introduction

Chronic airway diseases are major causes of human mortality (1, 2), represented by conditions such as cystic fibrosis (CF), chronic obstructive pulmonary disease (COPD), bronchiectasis, and asthma. Key features of these diseases are recurrent lower respiratory bacterial infections, which over repeated courses of antibiotic therapy can become antimicrobial resistant (AMR). Across these lung conditions, neutrophilic inflammation sustained by frequent infections can be a major driver of airway damage (3), yet effective anti-inflammatory and neutrophil-targeted therapies are not available.

An emerging tool against AMR infections is the therapeutic use of bacteriophages or phages, viruses that infect bacteria, ubiquitously and abundantly present in the environment (4). First identified in the early twentieth century, phages were initially investigated for use as antimicrobials in humans following observations of bacterial killing *in vitro* (5). Upon the discovery of penicillin and other antimicrobial compounds in the 1940s, interest in phages waned as academia and industry focused on development of these drugs; however, therapeutic phage centers have remained active in certain countries (5). With the emergence of antimicrobial resistance, and four decades since the last antibiotic drug was discovered, there is resurging interest in phages as potential alternatives. Phage therapy works by exploiting the life cycle of lytic phages, which in the process of replication lyse and kill their bacterial host (5). Phages have several characteristics that support their clinical use. They do not infect human cells (5), appear to be safe and well tolerated (6–8), may require less doses compared to conventional antibiotics due to their self-replicating nature (9, 10), and are highly specific to their target bacterial species, meaning unlike antibiotics they do not have broad bactericidal activity against the host microbiome (7, 11). The use of phages as a standard clinical therapeutic is hampered by a still incomplete understanding of phage biology (12), as well as a lack of regulatory manufacturing guidelines for phage products (13, 14) and standardized large-scale clinical trials (15); however, science, medicine, and industry are progressively working to surmount these challenges. In the context of chronic respiratory diseases, phage therapy is now being explored as a treatment for pulmonary infections (16, 17). Intriguingly, emerging evidence suggests that administration of phages may also have significant therapeutic benefits for managing neutrophilic inflammation in the lungs (18–21).

## The role of bacterial infections in chronically diseased airways

The link between bacterial airway infections and chronic inflammatory lung diseases is well established. One significant bacterium in this regard is the opportunistic pathogen *Pseudomonas aeruginosa*, a species which is notably associated with severe and negative health outcomes across multiple chronic airway conditions (22). Perhaps most striking is the early childhood acquisition in the autosomal recessive disorder CF, where *P. aeruginosa* contributes to reduced lung function (23–26), increased airway inflammation (27, 28), permanent airway remodeling (29, 30), and increased mortality in individuals with the disease (31, 32). Treatment strategies initiating eradication therapy in children with CF have reduced *P. aeruginosa* colonization rates from 80 to 50% (33, 34), but acquisition of this bacterium remains a key determinant of long-term CF clinical outcomes (31, 35). Among individuals with COPD, up to 40% will have positive sputum cultures for *P. aeruginosa* (36–39), with over 10% meeting criteria for colonization (39–41). In addition, up to a third of participants in cohorts of non-CF bronchiectasis can be colonized by this pathogen (42–44). The degree to which *P. aeruginosa* colonization in non-CF airway diseases contributes to lung function decline is still not clear (45), but multiple studies in both COPD and bronchiectasis link *P. aeruginosa* to more frequent exacerbations and/or hospitalization (36, 38, 42, 44, 46–48), increased mortality (36, 44, 46, 47, 49, 50), and greater annual lung function decline (40).

The role of bacterial infections in the pathogenesis of asthma is not as well understood as that of respiratory viruses, which are associated with childhood wheezing, compromised epithelial barrier function, asthma development, and exacerbations (51, 52). However, studies have still noted associations between bacterial pathogens and asthma pathologies. For example, in a cohort of 56 asthmatic patients from Royal Brompton Hospital, London, sputum bacterial culture positivity with *P. aeruginosa*, *Haemophilus influenzae*, and *Staphylococcus aureus* was significantly associated with increased asthma duration and frequency of exacerbations in the previous year (53). Other factors including pneumonia, pathogen isolation, as well as sputum production and purulence have also been identified and associated with the development of bronchiectasis in asthma cohorts (54, 55).

A primary concern with treating frequent lung infections in the context of these diseases is the acquisition of antimicrobial resistance, with some pathogens becoming multi-drug resistant (MDR). This makes eradication of these bacterial infections challenging and increases the treatment burden of patients with chronic lung conditions. In 2019, lower respiratory infections globally accounted for over 1.5 million out of 4.95 million estimated deaths associated with antimicrobial resistance, more than any other infectious syndrome (56). Among individuals with chronic lung diseases, acquisition of AMR/MDR pathogens is associated with increased disease severity (57–59), exacerbations (57, 59, 60), and mortality (31, 61). With prevalence of chronic airway conditions increasing by nearly 40% since 1990 (62, 63), novel therapeutics to treat AMR lung infections are desperately needed.

## Neutrophils, drivers of lung damage

As one of the first immune cell types recruited to sites of infection, neutrophils have an important role in the innate immune response to respiratory bacterial infections (64). Historically perceived as functionally rigid and transcriptionally fixed, neutrophils are increasingly described as plastic cells whose function is shaped by their environment (65–67). In the context of chronic lung diseases, studies assessing neutrophils recruited to diseased airways have observed changes in neutrophil antimicrobial functions that result in airway damage and contribute to lung disease progression.

One of the major mechanisms by which neutrophils can damage the airways is through release of neutrophil elastase (NE), a serine protease normally stored within intracellular primary granules. Airway NE is an important marker of inflammation in CF, significantly correlating with severity of lung disease in both children and adults (68, 69). Infection with *P. aeruginosa* is associated with increased sputum NE activity in adults with CF (70), as well as prolonged NE activity in pediatric CF airways (28). Release of NE by neutrophils in CF airways was originally thought to be a consequence of neutrophil death, but studies within the last decade have described how this process occurs from granule exocytosis by viable neutrophil populations in CF lungs (71–73). Work by our group using *in vitro* modeling of the airway infection environment created by *P. aeruginosa* has demonstrated that infection induces neutrophil degranulation (74). We found that neutrophils recruited to infection microenvironments primed by *P. aeruginosa* had significantly increased staining of CD63 and CD66b, neutrophil markers of primary and secondary granule exocytosis, respectively (74). In COPD, airway NE is elevated during exacerbations and can be predictive of bacterially induced exacerbations (75). Studies are also identifying airway NE as a potential biomarker of disease severity in non-CF bronchiectasis. For example, in a cohort of 433 adult patients, Chalmers and colleagues found significant associations between high sputum NE levels and increased dyspnea scores, lung function decline, exacerbation frequency, and radiological scoring of bronchiectasis severity (76). Sputum NE was elevated during exacerbations and reduced in response to antibiotic therapy targeting organisms such as *P. aeruginosa* and *H. influenzae*, highlighting a relationship between release of NE and bacterial respiratory infection (76). Another recent pediatric cross-sectional study of both CF and non-CF related bronchiectasis also found that sputum NE significantly correlated with exacerbation severity and frequency, as well as number of hospitalizations (77). For CF bronchiectasis it was specifically observed that NE correlated with risk of disease progression and increased lung function decline, while for non-CF bronchiectasis, sputum NE positively correlated with airway neutrophil counts and severity of lung disease (77). Asthma is a chronic lung disease with a much broader range of inflammatory phenotypes, and while eosinophil activity is critical to some cohorts, some of the more severe forms of asthma are primarily a result of airway neutrophilic inflammation (78). Past studies of asthma have associated increased airway NE (79–81) and myeloperoxidase (82), another factor released from primary granules, with more severe disease. Studies on allergic asthmatic responses in animal models have also shown reduced airway inflammation (83, 84) and bronchoconstriction (85) following treatment with NE inhibitors.

Neutrophil degranulation and NE release seem to coincide with a reduction in phagocytic ability that, certainly in CF, may contribute to further disease (71–74, 86). There are few reports describing decreased phagocytosis by neutrophils in COPD (87–89), bronchiectasis (90, 91), and asthma (92), and further investigation is required to definitively conclude whether this is a feature of non-CF lung diseases. Impairment of this crucial neutrophil function may contribute to prolonged infection, pathogen colonization, and associated negative health outcomes in chronic airway diseases, and may explain in part why neutrophils in these conditions resort to alternative antimicrobial strategies detrimental to host airways.

In the last 20 years, a novel neutrophil antimicrobial function was discovered and linked to the production of neutrophil extracellular traps (NETs) (93). This was termed NETosis, a process in which neutrophils eject extracellular networks of DNA containing primary granules, NE and other antimicrobial factors, which can trap and neutralize invading pathogens. Also thought to be an event resulting in neutrophil death (94), different NETosis pathways have been described that utilize mitochondrial DNA release rather than nuclear DNA (95), or preserve neutrophil viability after NET formation (96, 97). The degree to which NETs significantly contribute to pathogen clearance is debated (98, 99). The toxic antimicrobial factors harbored within NET complexes may instead contribute to airway damage. Interest in NETs has increased since studies identified them as major sputum components in chronically disease airways (100–102). In CF, NETs influence airway mucus viscosity (100, 103) and are associated with increased airway obstruction (104). CF neutrophils are also inherently predisposed to increased NET formation, delayed apoptosis and increased lifespan as a result of CFTR dysfunction (105). NET formation in COPD sputum has been found to significantly correlate with disease severity, lung function decline, and exacerbation frequency (101, 106). In severe asthma, high extracellular DNA indicative of increased NETosis has been associated with increased corticosteroid use, neutrophilic inflammation, and inflammasome activation (107). An international observational study by Keir et al. performed proteomic analysis of sputum from bronchiectasis patients, finding that NET proteins were abundantly present and strongly associated with increased disease severity, hospital admissions, and mortality (102). A separate study within this report further showed that low doses of antibiotics over a 12-month period was associated with NET reduction in sputum from individuals with bronchiectasis or asthma (102), underscoring the connection between NETosis and airway bacteria.

**FIGURE 1:**
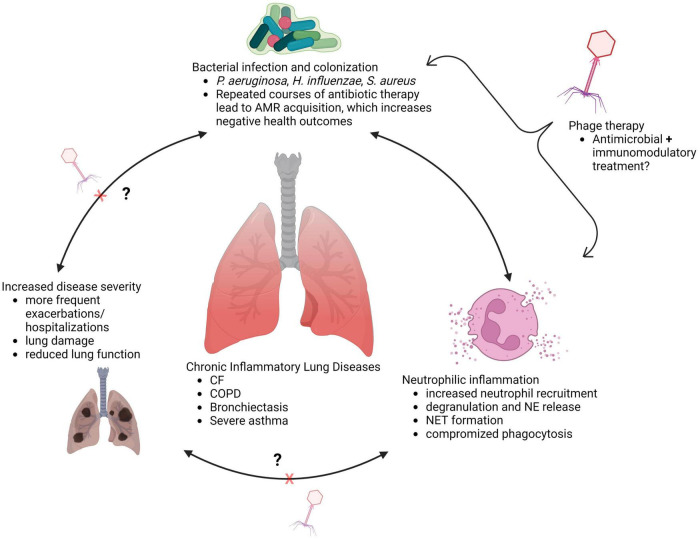
Interplay between infection and inflammation in chronically diseased lungs (created with BioRender.com).

Limiting neutrophil migration to the lungs would appear to be a simple solution for preventing damage by aberrant functions of recruited neutrophils. However, reducing airway neutrophil influx can have negative consequences, as was the case in a phase two clinical trial of the leukotriene B4 (LTB4) receptor antagonist BIIL 284 BS (108). This trial was prematurely terminated upon discovering a significant increase in serious adverse events among CF patients receiving the drug (108). A follow up study assessing participant samples and BIIL 284 treatment in *P. aeruginosa* infected mice determined that the drug significantly reduced airway neutrophil counts, leading to increased *P. aeruginosa* in the lung, bacteremia, and increased lung inflammation (109). This suggests that outright reduction of neutrophil numbers in infected lungs is not therapeutically beneficial; a therapy that instead amends neutrophil pathological activity, preserves phagocytic function, and aids in bacterial clearance, may be more effective. In chronic lung diseases, the interplay between bacterial respiratory infections, neutrophilic inflammation, and airway damage highlights an important need for therapies that can treat infection, and the inflammatory processes and neutrophil functions that result in damage to the lungs (Figure 1).

## Phage therapy and airway inflammation

Trials of phage therapy for respiratory infections in humans have occurred in a limited number of instances for compassionate use, particularly in cases with MDR pathogens where the traditional spectrum of antibiotics failed to clear infection. Many of the resulting case reports have described positive outcomes, with no adverse effects and infections successfully cleared in treated patients (110–115). However, despite increasing validation for use as antimicrobials in humans, little is known about the innate immune response to respiratory phage therapy, and how airway neutrophils recruited during bacterial infection may respond to treatment. A handful of studies in small animal models have provided some data on inflammation following therapeutic phage administration during experimental airway infection (20, 116–119).

Airway neutrophil recruitment is initiated by detection of chemotactic signals such as interleukin (IL)-8 and LTB4 (120). As neutrophils exit circulation and migrate through tissue, they can encounter additional inflammatory cytokines such as IL-1, IL-6, IL-18, TNFα, and become primed, further increasing their responsiveness and propensity for pathological activation (67, 121). Thus, host cytokines have an important role in modulating neutrophil responses during infection. A study by Pabary et al. assessed the effects of an intranasally administered mixture of individual phages or phage cocktail, before, during, and after *P. aeruginosa* inoculation in mice, measuring inflammatory markers and neutrophil counts in bronchoalveolar lavage fluid (BALF) (116). In experimental infections with *P. aeruginosa* reference strain PAO1, phage treatment at all timepoints significantly reduced viable bacterial numbers, but only the prophylactic administration of phages significantly reduced BALF neutrophil counts compared to untreated animals (116). Simultaneous inoculation with phages and bacteria significantly reduced IL-10 and IL-1β compared to animals infected with bacteria alone, while both delayed and prophylactic administration of phages significantly reduced the neutrophil chemokine keratinocyte chemoattractant (KC) (116). A CF clinical isolate of *P. aeruginosa* was also tested, inoculated simultaneously with lytic phages. Curiously, bacterial clearance of this isolate was not enhanced with phage treatment compared to untreated animals; however, the authors acknowledged differences in bacterial doses and BALF sampling times in experiments with the clinical isolate vs. PAO1 that may account for this (116). Nevertheless, phage treatment in this experiment significantly reduced both neutrophil counts and pro-inflammatory mediators IL-6, IL-10, IL-12p70, KC, and TNFα in BALF of treated animals compared to untreated controls (116). A different study using bioluminescent *P. aeruginosa* was able to image phage-mediated clearance in the lungs of infected mice, with treatment with phages reducing bacterial luminescent signal from the lungs, increasing animal survival, and reducing IL-6 and TNFα in BALF (117). It was also determined that prophylactic phage administration twenty-fours prior to bacterial inoculation had a protective effect against infection (117). Importantly, a study of *Escherichia coli* pneumonia in mice showed that bacterial lysis induced by phage therapy induces similar levels of cytokine release as lysis induced by antibiotics, with phages primarily reducing release of most inflammatory signals (118). This suggests phage-induced bacterial lysis is unlikely to result in more severe inflammation compared to activity of conventional antibiotics, but additional research is needed to verify this.

While by design not a respiratory model, a *CFTR* loss-of-function zebrafish model has been used by Cafora et al. to describe the immunomodulatory potential of phage therapy in CF across two studies. In the first, a phage cocktail administered to zebrafish embryos infected with PAO1 was observed to significantly reduce bacterial load, lethality, and gene expression of IL-1β and TNFα (119). Of note, reduced cytokine gene expression was also observed in embryos exposed to phages alone in the absence of bacteria, suggesting phage anti-inflammatory mechanisms independent of bactericidal activity (119). This was a major focus of the second study, which identified that embryo toll-like receptor (TLR) recognition of phage capsid proteins, and not phage DNA, was necessary to elicit an anti-inflammatory effect (20). The injection of a phage cocktail at the site of experimental tail amputation was further observed to reduce neutrophil recruitment to wound sites, further demonstrating phage capacity to influence localized inflammation (20).

As a direct barrier to infection, the airway epithelium is a major source of neutrophil chemotactic signals and inflammatory mediators (122, 123). In perhaps one of the only studies assessing effects of phage therapy on primary airway epithelial cells, Trend and colleagues performed exposures of undifferentiated primary airway epithelial cell cultures to the virulent *P. aeruginosa* phage E79 (124). They observed that E79 did not increase release of pro-inflammatory cytokines IL-1β, IL-6, IL-8, or induce apoptosis, in airway cultures derived from children with and without CF (124), indicating that phages alone are not highly immunostimulatory to human airway cells. Altogether, studies suggest that phage therapy can effectively reduce cytokine signals involved in neutrophil recruitment and activation. This effect is not always a consequence of overt antimicrobial activity, with induction of anti-inflammatory mediators (125), reduced production of reactive oxygen species (126), and LPS binding (127) identified as possible mechanisms. This would make phages an attractive multipurpose therapeutic for managing both airway inflammation and infection in chronic lung diseases. However, further investigation is necessary to understand the specific mechanisms of phage anti-inflammatory activity.

## Phage-neutrophil interactions

The interactions between phages and human phagocytes have been of interest to researchers since the 1920s, when a number of early studies noted increased phagocytosis of bacteria by leukocytes in the presence of phages (128–130). More recent studies in neutropenic mouse models have noted a synergism between phages and neutrophils that is required for successful clearance of bacteria. An investigation by Tiwari et al. found that immunocompetent mice inoculated intranasally with a lethal dose of PAO1 could clear lung infection and maintain an 80–100% survival rate when receiving different doses of lytic phages; however, neutropenic animals failed to clear infection with phage administration alone (131). Roach and colleagues took this approach a step further, using *in silico* modeling to identify host innate responses as a necessary feature to overcome emerging phage resistant mutants during respiratory *P. aeruginosa* infection, and neutrophil activity as a key component of successful phage therapy (21). Whether this synergy implies phage-mediated enhancement of neutrophil bacterial killing capacity is an important question for future research. Some studies have linked phages to increased intracellular killing of pathogens within human phagocytes such as *Klebsiella pneumoniae* (132), *Mycobacterium avium* and *tuberculosis* (133), and methicillin-resistant *S. aureus* (134, 135). There are contrasting reports, however, where phages did not significantly influence intracellular killing of pathogenic bacteria (136, 137).

Whether phages contribute to activation of pathological neutrophil functions is critical to ascertain for safe use of this therapy. A study of neutrophil exposure to lytic phages observed little to no respiratory burst activity induced by T4 *E. coli* phage and A3/R *S. aureus* phage preparations, compared to heat inactivated *S. aureus* cells, suggesting that phages alone should not induce oxidative stress when administered in humans (19). Importantly, it has also been shown that A3/R phage and *S. aureus* phage lysate do not elicit neutrophil degranulation, as indicated by low neutrophil expression of CD63 and CD66b (18). This implies that both phages and phage-mediated lysis of bacteria are not likely to activate neutrophil degranulation and consequent NE release during treatment *in vivo*. However, availability of data in this regard is inconsistent across bacterial pathogens and neutrophil activation states, so there remains much work to be done for a definitive understanding. Further exploration of whether phage therapy can restore neutrophil phagocytosis of evasive organisms and ameliorate aberrant functions such as degranulation is warranted, particularly in the context of inflammatory airway diseases.

## Modeling phage therapy and neutrophilic inflammation in the laboratory

**FIGURE 2:**
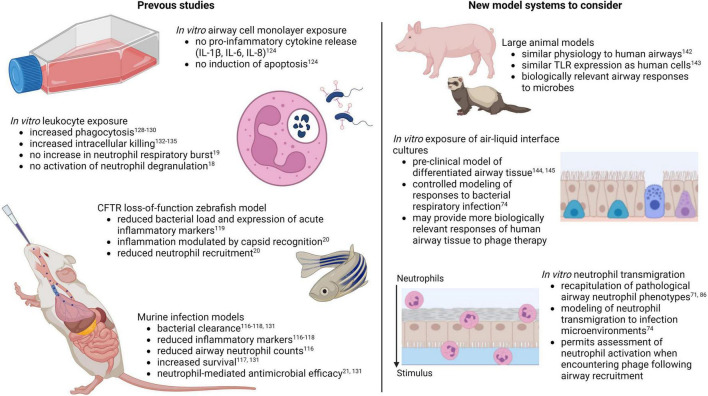
Models for assessing innate immune responses to pulmonary phage therapy (created with BioRender.com).

Altogether, studies to date suggest potential anti-inflammatory and neutrophil-modulating benefits of phage therapy for respiratory infections. Further research on this topic is justified, as findings could point to novel therapeutic benefits with capacity to improve treatment of multiple chronic inflammatory lung conditions. However, several factors must be taken into account for relevant modeling of human airway immune responses during phage therapy. For the purposes of understanding airway cell and neutrophil responses to pathogen associated molecular patterns during infection, a major limitation of murine models is differential TLR expression. Mice contain a pseudogene for TLR10, an anti-inflammatory TLR shown to detect bacterial and viral ligands, which is normally expressed by human cells (138). Furthermore, mice and rats express three TLRs that are not express in humans, TLRs 11, 12, and 13, which detect flagellin, fungal profilin, and bacterial ribosomal sequences, respectively (139). In addition, mouse neutrophils may not be activated by certain microbial factors that normally affect human neutrophils, as has been reported with staphylococcal superantigen-like protein 13 (SSL13) (140). These limitations can be overcome by using large animal models of chronic airway disease including pigs and ferrets (Figure 2) (141, 142), whose airway physiology and TLR expression more closely resemble that of human airways (143).

A major drawback of large animal models is the high cost, labor, and resources required. This is where cell-based laboratory models have an advantage. Few studies to date have assessed phage safety and efficacy on cultures of differentiated primary airway epithelial cells known as air-liquid interface cultures (Figure 2) (144, 145), the gold-standard model for pre-clinical studies in human airways. Further research in this model may provide valuable and biologically relevant insights on innate responses to phage therapy in human lungs. Regarding *in vitro* studies of phage-neutrophil interactions, one of the major caveats of previous studies is the restricted exposure of phages to peripheral blood neutrophils. This may provide insights into how neutrophils in circulation interact with phages delivered by intravenous injection, but it fails to account for the fact that the site of infection and the extravasation process itself contribute to pathological neutrophil activation (66, 67). Granule releasing neutrophils in CF, as an example, are only evident in the airway lumen, as peripheral blood neutrophils in individuals with CF are phenotypically similar to neutrophils from non-CF individuals (71, 73). Furthermore, recapitulation of this neutrophil phenotype in the laboratory can only be achieved following *in vitro* transmigration (71, 86). Existing laboratory models of neutrophil recruitment to the lungs may provide more relevant examples of neutrophil behavior during respiratory phage therapy, as neutrophil responses to phages can be observed following transmigration to the airway infection environment (Figure 2).

## Conclusion

In summary, neutrophils are important and necessary for the clearance of bacterial respiratory pathogens. In chronic and inflammatory airway diseases, persistent bacterial infections sustain neutrophil influx into the lungs, wherein exaggerated neutrophil antimicrobial functions can result in host tissue damage. Phage therapy is emerging as novel therapeutic for AMR lung infections resulting from prolonged antibiotic use. A growing body of evidence suggests phage therapy may have important immunomodulatory benefits. Whether this is primarily a consequence of reduced bacterial burden or direct interaction between phages and neutrophils merits further investigation. Various laboratory model systems are available to assess airway innate responses to phage therapy; researchers must ensure that models are representative of these dynamics in human airways.

## Author contributions

DL, LG, and AK conceived the review, conduced the literature search, and wrote the manuscript. SS critically reviewed and edited the manuscript. All authors approved the final version.
